# Aging Makes the Heart Grow Fonder: Age Influences Hearing Ability and Interactions between Psychological Phenomena in Patients with Chronic Tinnitus

**DOI:** 10.3390/jpm14010023

**Published:** 2023-12-23

**Authors:** Maren Fresemann, Benjamin Boecking, Kurt Steinmetzger, Petra Brueggemann, Matthias Rose, Birgit Mazurek

**Affiliations:** Tinnitus Center, Charité—Universitätsmedizin Berlin, Charitéplatz 1, 10117 Berlin, Germany; maren.fresemann@charite.de (M.F.); benjamin.boecking@charite.de (B.B.); kurt.steinmetzger@charite.de (K.S.); petra.brueggemann@charite.de (P.B.); matthias.rose@charite.de (M.R.)

**Keywords:** age, tinnitus-related distress, hearing ability, psychological distress

## Abstract

(1) Background: Risk factors for chronic tinnitus comprise interactions of individuals’ hearing difficulties and psychological distress—including anxiety, depression, and perceived stress levels. Both groups of factors likely become more pronounced with age, although mixed literature has also suggested increases in psychological resilience over time. To this end, only a few studies have delineated direct and indirect effects of age on audiological and psychological variables that might influence tinnitus-related distress in patients with chronic tinnitus. (2) Methods: *N* = 678 patients with chronic tinnitus completed audiological assessments alongside self-report measures of psychological and tinnitus-related distress. Path analyses investigated the effect of age on patients’ tinnitus-related distress via both audiological and psychological variables. (3) Results: Age was significantly associated with both hearing ability and psychological distress indices, with perceived stress and anxiety decreasing with aging. Different mediation models revealed that the association between age and tinnitus-related distress was mediated completely by hearing ability and partly by perceived stress and anxiety. (4) Conclusions: Whilst interactions of individuals’ hearing ability and psychological distress levels maintain tinnitus-related distress across the age span, the weighting of these factors may shift with age. Treatment approaches should consider hearing ability across the lifespan. Psychological factors should be individually conceptualized, considering both distress-related and potential resilience factors in old age.

## 1. Introduction

As the population ages, age-related hearing loss (presbycusis) is becoming an increasingly serious issue [1]. Hearing difficulties are the most common sensory deficit in older age and are associated with reduced quality of life and heightened feelings of isolation, dependency, and frustration [1]. Studies dealing with hearing ability have focused mainly on hearing loss as well as its effects. According to the WHO [2], pure tone average (PTA) values higher than 26 dB (based on hearing thresholds) in the better ear can be considered as hearing loss. Research at the junction of hearing loss and psychological phenomena has examined (1) differences in anxiety and depression in individuals with or without hearing loss, (2) associations between the degree of hearing loss and psychological phenomena, and (3) the predictive value of hearing loss for the subsequently assessed anxiety (or vice versa). Studies have reported mixed results. There was a higher prevalence of anxiety [3] and a higher risk of developing depression [4] in people with hearing loss compared to people without hearing loss. Moreover, anxiety and depression have been reviewed and found in studies to be a possible consequence of hearing loss [5,6,7,8,9]. Hasson et al. [10] found occupational stressors as well as more symptoms of long-lasting stress to be significantly associated with a higher prevalence of hearing problems. Another study found that perceived stress predicted sensory difficulties with both hearing and vision three years later and moreover mediated the relation between depression and hearing [11]. Other studies have found hearing loss to be associated with previous anxiety disorders [12,13].

Hearing loss is also associated with tinnitus symptom onset [14]. In fact, it denotes the only established ontological risk factor for chronic tinnitus [15]. Whether the tinnitus symptom is then being interpreted as distressing depends on the subsequent psychological processing of the symptom [16,17]. Basso et al. [18] found associations between bothersome tinnitus and reduced hearing ability, as well as hearing-related difficulties. Pinto et al. [19] found no effect between tinnitus annoyance, age, and hearing loss. Heterogeneous findings can be explained due to the differentiation of hearing loss and tinnitus onset on the one hand, and hearing loss and tinnitus-related distress on the other hand.

Hearing ability worsens across the age span [1]. Prevalence rates showed 26.8% hearing loss (<25 dB) in the age span of 60–69 years, 54–55% in the age span of 70–79 years, and 79–81% in people older than 80 years [20].

Researchers have postulated that chronic tinnitus (“the conscious awareness of a tonal or composite noise for which there is no identifiable corresponding external acoustic source” [21], p. 1) may constitute a phenomenon that is largely independent from acute, medically mediated tinnitus presentations. Therefore, it is important to distinguish between the tinnitus symptom and tinnitus-related distress—which likely stems less from symptom characteristics “per se”, but more from individuals’ psychological appraisal and experience of the tinnitus symptom within idiosyncratic psychological life contexts. Indeed, psychological influences such as depression and anxiety appear to underlie the chronification process and predict the ongoing perception of the symptom within vicious cycles spanning pre-existing emotional distress, processing of the tinnitus symptom, and tinnitus-related distress [22].

In chronic tinnitus presentations, the crucial roles of depression, stress, and anxiety have been frequently highlighted—although causal and consequential relationships are difficult to distinguish. Stegeman et al. [23] found higher levels of stress, depression, and anxiety as significant risk factors for people suffering from tinnitus. Kim [24] found a direct relationship between tinnitus-related distress and the level of emotional distress. Probst et al. [25] found that the relationship between subjectively reported tinnitus loudness and tinnitus-related distress was mediated by emotional distress. Especially, depression and anxiety are common amongst tinnitus patients and conceptually overlap with tinnitus-related distress [14,24,26,27,28,29,30,31,32,33].

Psychological distress may also worsen with age, although the literature in this regard is heterogeneous. For example, Jokela et al. [34] reported that the risk of common mental disorders appeared to increase with age—especially in older people above age 75. Levels of perceived stress increased after the age of 65 [35]. Depression and anxiety were found to be highly prevalent among people aged 90 years old [36]. Other studies found opposite effects. Gondek et al. [37] found that psychological distress levels increased between early adulthood and midlife and decline thereafter. Reynolds et al. [38] examined individuals above age 55 and found a decrease in psychiatric disorders with increasing age.

As a non-modifiable, superordinate direct or indirect risk factor, ‘age’ has recently been examined in all three variable groups: (1) hearing loss, (2) tinnitus symptom onset, and (3) chronic tinnitus/psychological factors. Whilst the risk of hearing loss [20] and hearing-loss associated tinnitus symptomatology [39] seem to increase with age, psychological variables seem to change in a more mixed manner across the lifespan. Some studies found a deterioration in mental health with aging [34,35], while others found an improvement [37,38,40].

Regarding age-associated influences on tinnitus symptom and tinnitus-related distress, associations between age, tinnitus symptom prevalence, and tinnitus-related distress are heterogeneous and are likely influenced by audiological as well as psychological third variables, the prevalence of which may also increase or vary with age.

Several studies compared the prevalence of self-reported tinnitus symptoms across different age groups. Whilst some studies reported increasing prevalence rates with increasing age [41,42], other studies failed to find any such differences [31,43].

A similar picture emerges from studies examining the connection between age-associated influences and tinnitus-related or psychological distress. According to Milerova et al. [27], increasing age was associated with higher tinnitus-related distress. Gibrin et al. [44] reviewed relations between tinnitus symptoms, tinnitus-related distress, anxiety, and depression in the elderly and found age to contribute to the increase of tinnitus-related distress and depression and anxiety. They found that chronic tinnitus was subjectively louder, more annoying, and more distressing in older patients compared with younger patients. This suggests that the tinnitus symptom is more likely to be interpreted in an anxiety-inducing manner in older age groups where overall psychological distress levels have been found to be increased in some studies [34,35], but not others [37,38,40].

Kim et al. [42] found the proportion of “uncomfortable” tinnitus increased with age. They discussed difficulties of adapting to tinnitus in older age, which might be influenced by other psychological factors.

In contrast to the aforementioned findings, Park et al. [45] found no significant differences between younger and older patients in tinnitus-related distress, depression, and stress scores. Al-Swiahb and Park [41] found no significant differences in tinnitus-related distress between age groups, but higher amounts of catastrophic tinnitus in the age group older than 60 was observed.

Associations between age, the prevalence rates of hearing loss, and tinnitus-related distress, as well as psychological distress or “disorders” are likely complex. Within this field, however, no study has explicitly linked age with (a) hearing ability, (b) tinnitus-related distress, and (c) other psychological variables. The present study aimed to fill this gap by investigating these relations. We hypothesized that

1. Increasing age is associated with
  - decreased hearing abilities;
  - tinnitus-related distress;
  - perceived stress, anxiety, and depression.
2. As suggested by Kim et al. [42], we hypothesized that
  - a relationship between age and tinnitus-related distress is mediated by perceived stress, anxiety, depression, and hearing ability.

## 2. Method

### 2.1. Participants

The present study included *N* = 678 self-referred patients who were treated at the Tinnitus Centre, Charité Universitatsmedizin, Berlin, Germany. Data were collected between January 2019 and December 2020. Patients suffered from chronic tinnitus (lasting longer than three months), were at least 18 years old, and completed German versions of the Tinnitus Questionnaire, Perceived Stress Questionnaire, and the Hospital and Anxiety Scale, among other scales, as part of the initial diagnostics. Patients further underwent pure-tone audiometry procedures to assess hearing abilities as part of the initial routine diagnostic procedures. Exclusion criteria comprised the presence of acute psychotic illness or addiction, (untreated) deafness, and insufficient knowledge of the German language. Participants gave written consent for the data to be collected and used for research purposes. The Charité Universitatsmedizin Berlin’s ethics committee approved data collection and analysis (No: EA4/216/20).

### 2.2. Procedure

All analyses were conducted using IBM SPSS Statistics 29. In the beginning, a plausibility analysis identified implausible or missing values. Pearson’s correlation coefficient r examined associations between continuous variables. According to Cohen [46], correlation coefficients could be interpreted as r = 0.10 (small effect), r = 0.30 (medium effect), and r = 0.50 (large effect). We calculated means of hearing ability (PTA4) and age groups referring to lifespan development by Erikson [47]. The latter were used to exemplify demographic variables (Table 1). All mediation analyses were conducted using PROCESS macro for SPSS (Version 4.0). Due to slight violations of homoscedasticity in the dependent-variable hearing ability (PTA4), BCa-Bootstrapping (1000 samples) was conducted in the first regression model (4.2.1) and robust standard estimators (HC3) were used for calculating all mediation models.

To investigate the role of age, we conducted three sets of analyses:

First, separate linear regression analyses investigated the effect of age on (a) hearing ability, (b) tinnitus-related distress, (c) perceived stress, (d) anxiety, and (e) depression.

Second, simple mediation models examined the indirect effects of (a) age on tinnitus-related distress via significant dependent variables from the previous analysis.

Third, multiple mediation models examined indirect effects of (a) age on tinnitus-related distress via combinations of significant mediators from the previous analysis.

## 3. Materials

### 3.1. Audiometry

Audiological testing included pure-tone audiometry, speech intelligibility in noise tests, and loudness discomfort levels. Hearing ability was assessed by measuring hearing thresholds for frequencies across 0.5, 1, 2, and 4 kHz and separately averaging them (PTA) for both ears. Decreased hearing abilities were reflected with an increase of PTA.

### 3.2. Questionnaires

#### 3.2.1. Tinnitus Questionnaire (TQ, German Version)

The Tinnitus Questionnaire [48] measures tinnitus-related distress with self-reporting. The German Version [49] consists of 52 different items, 40 items of which are answered on a 3-point-likert scale (0 = not true, 1 = partly true, and 2 = true), and two items of which are counted twice, thus yielding a total score range between 0 and 84 points. The German Version has been validated. Test–retest reliability was r_tt_ = 0.94 for the total score. The internal consistency for the total score was α = 0.94 [49]. In the current study, the internal consistency of the total score was excellent with α = 0.94.

#### 3.2.2. Perceived Stress Questionnaire-20 (PSQ-20, German Version)

The Perceived Stress Questionnaire-20 [50] is a self-reporting measure that assesses subjectively perceived stress experiences. The current study used the German Version [51]. It consists of 4 subscales (worries, tension joy, and demands), 5 items of which are answered on a 4-point-likert scale (1 = hardly ever, 2 = sometimes, 3 = often, and 4 = usually). A total score is computed by linearly transforming and averaging all items to range from 0 to 100. The total score’s internal consistency has been reported to fall between 0.80 and 0.86 [50]. The internal consistency in the current study was excellent with α = 0.94.

#### 3.2.3. Hospital Anxiety and Depression Scale (HADS, German version)

The German version [52] of the Hospital Anxiety and Depression Scale [53] is a 2-factor scale which measures patients’ levels of anxiety (HADS-A) and depression (HADS-D). Each scale consists of 7 items that are rated on a 4-point-scale with differing anchor points. Cronbachs α lies around 0.8 for both subscales [54]. Retest reliability was about r_tt_ > 0.8 (up to 2 weeks) [54]. The internal consistency in the current study was good with α = 0.8 for HADS-A and α = 0.88 for HADS-D.

## 4. Results

Patients were between 19 and 82 years old (*M* = 51.87 years; *SD* = 12.22) and 51.2% of the sample were female. Table 2 presents the means and standard deviations for all observed variables split by age groups, as well as significant group differences in the observed variables.

On average (+/−1*SD*), patients showed normal to moderate expressions in the Perceived Stress Questionnaire, caseness (normal-to-caseness) in the HADS-A and normal (normal-to-caseness) values in the HADS-D, and moderate expressions of tinnitus-related distress (mild-to-severe values).

### 4.1. Effects of Age

#### Simple Linear Regression Analysis: Effects of Age

**Figure 1:**
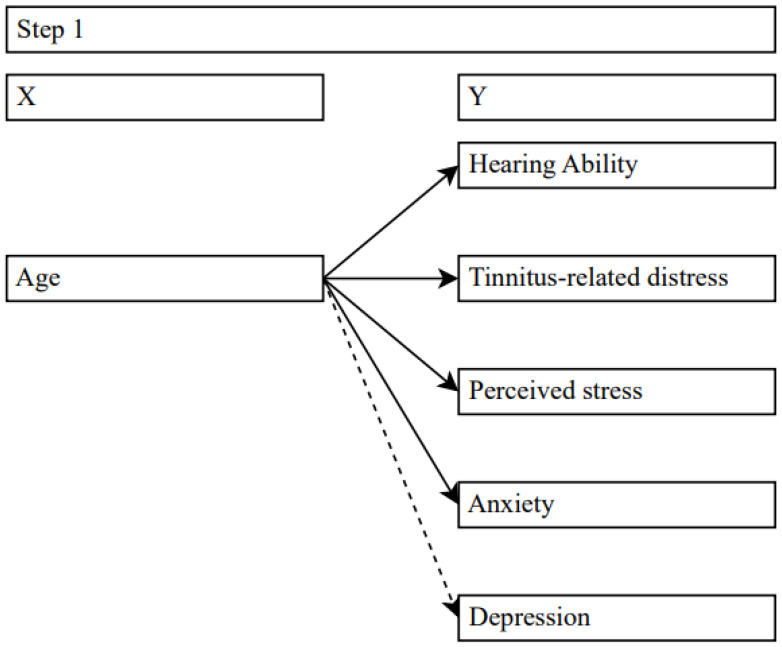
Effects of age on target variables. Solid lines = significant effects, dashed lines = no significance.

First, regression analyses examined the effects of age on hearing ability, tinnitus-related distress, other psychological variables, and depression (Figure 1).

Due to slight violations of homoscedasticity, the regression between age and hearing ability (PTA) was calculated with BCa-Bootstrapping (1000 samples). Age significantly predicted PTA (β = 0.45 [0.37; 0.49], *t*(676) = 12.91, *p* < 0.001). The model explained 20% of the variance (*R*² = 0.2, *F*(1, 676) = 166.64, *p* < 0.001) and supported our hypothesis that hearing ability decreases with increasing age.

Similarly, age predicted tinnitus-related distress (β = 0.1, *t*(676) = 2.65, *p =* 0.008) in that tinnitus-related distress slightly increased with increasing age. However, the model only explained a small part of the variance (*R*² = 0.01, *F*(1, 676) = 7.02, *p* < 0.012).

Age was negatively associated with perceived stress (β = −0.23, *t*(676) = −6.08, *p* < 0.001), explaining 5% of the variance (*R*^2^ = 0.05, *F*(1; 676) = 36.91, *p* < 0.001) and anxiety levels (*β* = −0.11, *t*(676) = −2.87, *p* = 0.004), where age explained only a small part of variance at 1% (*R*² = 0.01, *F*(1, 676) = 8.21, *p* = 0.004).

Regarding the relationship between age and depression, no significant effect emerged (β = 0.02, *t*(676) = 0.51, *p* = 0.614, *R*² = 0.00, *F*(1, 676) = 0.26, *p* = 0.614).

### 4.2. Simple Mediation Analyses: Effects of Age on Tinnitus-Related Distress via Hearing Ability, Perceived Stress, and Anxiety

**Figure 2:**
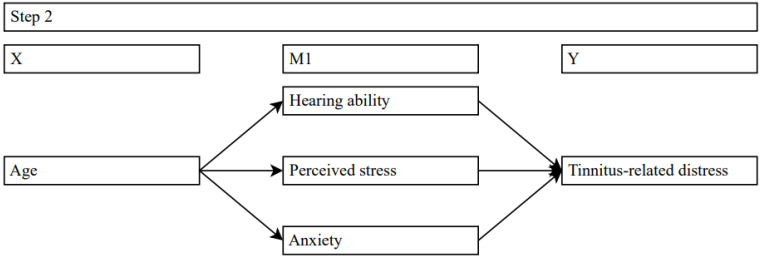
Simple mediation models examining effects of age on tinnitus-related distress via hearing- ability, perceived stress, and anxiety. Solid lines = significant effects.

Three different models examined the simple mediation effects of age on tinnitus-related distress via hearing ability, perceived stress, and anxiety (see Figure 2). Mediation models were calculated using bootstrapping (10,000 samples) and HC3 standard errors.

The first model included hearing ability (PTA) as a mediator on the relationship between age and tinnitus-related distress. Results indicated a significant positive relationship between age and PTA (*b* = 0.44, *SE* = 0.03, *t* = 14.62, *p* = 0.000) and between PTA and tinnitus-related distress (*b* = 0.32, *SE* = 0.6, *t* = 5.39, *p* = 0.000). Unlike the total effect (*b* = 0.14, *SE* = 0.05, *t* = 2.9, *p* = 0.004), the direct effect of age on tinnitus-related distress was not significant (*b* = −0.01, *SE* = 0.05, *t* = −0.09, *p* = 0.925). The indirect effect of age on tinnitus-related distress was found to be statistically significant (Effect = 0.14, *SE* = 0.03, C.I. [0.1; 0.19]). The results concluded that hearing ability completely mediated the relationship between age and tinnitus-related distress.

The second model specified perceived stress as a mediator for the relationship between age and tinnitus-related distress. The results revealed a significant *negative* relationship between age and perceived stress (*b* = −0.38, *SE* = 0.06, *t* = −6.49, *p* = 0.000) and a positive relationship between perceived stress and tinnitus-related distress (*b* = 0.41, *SE* = 0.03, *t* = 16.05, *p* = 0.000). The results further suggested that age continued to be associated with tinnitus-related distress once perceived stress was included in the model (*b* = 0.29, *SE* = 0.04, *t* = 6.73, *p* = 0.000). Compared to the total effect (*b* = 0.14, *SE* = 0.05, *t* = 2.9, *p* = 0.004), the direct effect between age and tinnitus-related distress increased when perceived stress was controlled. The indirect effect of age on tinnitus-related distress was found to be statistically significant (Effect = −0.16, *SE* = 0.03, C.I. [−0.21; −0.11]). These results showed that reductions in perceived stress partly mitigated the impact of age on tinnitus-related distress.

The third model examined specified anxiety as a mediator for the relationship between age and tinnitus-related distress. The results revealed a significant, though small negative relationship between age and anxiety (*b* = −0.04, *SE* = 0.01, *t* = −3.06, *p* = 0.002) and a strong positive relationship between anxiety and tinnitus-related distress (*b* = 2.31, *SE* = 0.12, *t* = 18.86, *p* = 0.000). The results further suggested that age continued to be associated with tinnitus-related distress once anxiety was included in the model (*b* = 0.22, *SE* = 0.04, *t* = 5.55, *p* = 0.000). Compared to the total effect (*b* = 0.14, *SE* = 0.05, *t* = 2.9, *p* = 0.004), the direct effect between age and tinnitus-related distress increased when anxiety was controlled. The indirect effect of age on tinnitus-related distress was found to be statistically significant (Effect = −0.09, *SE* = 0.03, C.I. [−0.14; −0.03]). These results showed that reductions in anxiety partly mitigated the impact of age on tinnitus-related distress.

### 4.3. Multiple Mediation Analyses: Effects of Age on Tinnitus-Related Distress via Hearing Ability and Perceived Stress; Hearing Ability and Anxiety; and via Perceived Stress and Anxiety

**Figure 3:**
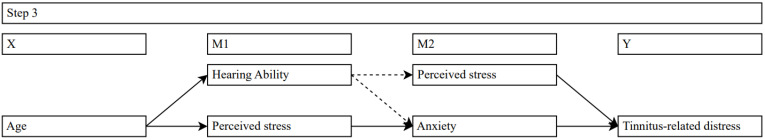
Serial multiple mediation models examining effects of age on tinnitus-related distress via interactions of hearing ability, perceived stress, and anxiety. Solid lines = significant effects; dashed lines = no significance.

Last, a multiple mediation model examined indirect effects of age on tinnitus-related distress via significant mediators from the previous analyses (hearing ability, perceived stress, and anxiety, see Figure 3). All mediation models were calculated using bootstrapping (10,000 samples) and HC3 standard errors.

The direct paths of the first model revealed a significant positive relationship between age and PTA (*b* = 0.44, *SE* = 0.03, *t* = 14.62, *p* = 0.000), no significant relationship between PTA and perceived stress (*b* = 0.09, *SE* = 0.07, *t* = 1.32, *p* = 0.189), and a significant positive relationship between perceived stress and tinnitus-related distress (*b* = 0.41, *SE* = 0.03, *t* = 16.19, *p* = 0.000). Neither the total indirect effect of age on tinnitus-related distress (Effect = −0.03, *SE* = 0.03, C.I. [−0.1; 0.04]) nor the indirect effect from age on tinnitus-related distress via PTA and perceived stress (Effect = 0.02, *SE* = 0.01, C.I. [−0.01; 0.04]) proved to be significant. Age continued to be associated with tinnitus-related distress once PTA and perceived stress were included in the model (*b* = 0.17, *SE* = 0.05, *t* = 3.44, *p* = 0.001). Compared to the total effect (*b* = 0.14, *SE* = 0.05, *t* = 2.9, *p* = 0.004), the direct effect between age and tinnitus-related distress slightly increased when PTA and perceived stress were controlled. The results concluded that interactions between PTA and perceived stress did not mediate the relationship between age and tinnitus-related distress.

The direct paths of the second model showed a significant positive relationship between age and PTA (*b* = 0.44, *SE* = 0.03, *t* = 14.62, *p* = 0.000), no significant relationship between PTA and anxiety (*b* = 0.03, *SE* = 0.02, *t* = 1.73, *p* = 0.084), and a strong positive relationship between anxiety and tinnitus-related distress (*b* = 2.26, *SE* = 0.12, *t* = 18.91, *p* = 0.000). Neither the total indirect effect of age on tinnitus-related distress (Effect = 0.03, *SE* = 0.02, C.I. [−0.0; 0.06]) nor the indirect effect of age on tinnitus-related distress via PTA and anxiety (Effect = 0.03, *SE* = 0.01, C.I. [−0.0; 0.04]) proved to be significant. Age continued to be associated with tinnitus-related distress once PTA and anxiety were included in the model (*b* = 0.11, *SE* = 0.04, *t* = 2.41, *p* = 0.016). Compared with the total effect (*b* = 0.14, *SE* = 0.05, *t* = 2.9, *p* = 0.004), the direct effect between age and tinnitus-related distress slightly decreased when PTA and anxiety were controlled. The results concluded that the interactions between PTA and anxiety did not mediate the relationship between age and tinnitus-related distress.

As for the direct paths in the third model, the results revealed a significant negative relationship between age and perceived stress (*b* = −0.38, *SE* = 0.06, *t* = −6.49, *p* = 0.000), a significant positive relationship between perceived stress and anxiety (*b* = 0.15, *SE* = 0.01, *t* = 28.88, *p* = 0.000), and a strong positive relationship between anxiety and tinnitus-related distress (*b* = 1.82, *SE* = 0.18, *t* = 10.19, *p* = 0.000).

The indirect effect of age on tinnitus-related distress via perceived stress and anxiety was also found to be statistically significant (Effect = −0.1, *SE* = 0.02, C.I. [−0.14; −0.07]).

The results further suggested that age continued to be associated with tinnitus-related distress once perceived stress and anxiety were included in the model (*b* = 0.26, *SE* = 0.04, *t* = 6.23, *p* = 0.000). Compared with the total effect (*b* = 0.14, *SE* = 0.05, *t* = 2.9, *p* = 0.004), the direct effect between age and tinnitus-related distress increased when perceived stress and anxiety were controlled. The total indirect effect of age on tinnitus-related distress was found to be statistically significant (Effect = −0.12, *SE* = 0.03, C.I. [−0.18; −0.06]).

The results supported our hypothesis that the relationship between age and tinnitus-related distress is mediated by perceived stress and anxiety. The interactions of individuals’ perceived stress and anxiety seemed to maintain tinnitus-related distress across the age span.

## 5. Discussion

The aim of this study was to examine age in its role for hearing ability, psychological variables, and tinnitus-related distress in a sample of patients with chronic tinnitus in routine clinical practice. The importance of this topic was also reflected in the large sample size and help-seeking in the population.

First, we examined whether age influenced hearing abilities, tinnitus-related distress, perceived stress, anxiety, and depression. We hypothesized that an increased age would be associated with a deterioration of hearing ability (synonymous with an increase in pure-tone average). The influence of age-related processes on hearing abilities, which has already been investigated many times [20,55], was also found in this study. There was a significant positive relation between age and PTA, meaning that hearing abilities decreased with rising age. The findings highlighted the importance of preventing hearing loss in younger age [56] or addressing hearing loss in older age using hearing amplification means, as applicable. Beyond hearing amplification, hearing aids may further positively impact psychological well-being by means of improved vitality, social functioning, emotional stability, and mental health [57], as well as a lower Odds Ratio of Major Depressive Disorder or any depressive symptoms [58].

Due to close connections between hearing loss and tinnitus symptom onset [43,59]—which is then, however, processed differently according to the psychological profiles of those affected—we hypothesized that an increased age would be associated with higher variance in tinnitus-related distress. Indeed, the results indicated that (a) an increased age was associated with higher levels of tinnitus-related distress; however, (b) this effect partly depended upon covarying perceived stress and anxiety levels.

Due to the crucial role of psychological factors in constituting tinnitus-related distress and maintaining chronic symptom perception, we further examined whether and how age would be associated with psychological distress levels, notably perceived stress, depression, and anxiety.

The results indicated that perceived stress levels and anxiety decreased with an increased age—with this effect directly benefitting tinnitus-related distress levels. Whilst the likelihood of hearing loss and, thereby, tinnitus symptom onset increased with age, the symptom’s psychological processing was largely unrelated to tinnitus “per se”, and psychological well-being denoted an important prevention target for ameliorating tinnitus-related distress in older age. One possible explanation for this effect may have been that increased life experience helps people develop more individually adaptive and fewer maladaptive coping strategies [60]. In this vein, Gooding et al. [61] found older adults to be more psychologically resilient (e.g., social support, emotional regulation skills, problem-solving abilities) than younger adults.

There was no significant effect of age on depression, meaning that depression scores were similarly distributed across the age span. This result contrasted with former studies, which found associations between age and depression in both directions [34,37,38,44]. However, the current sample yielded overall low levels of depression, and a possible floor effect might explain the observed non-relation.

Second, we hypothesized that the relationship between age and tinnitus-related distress is mediated by hearing ability (PTA), perceived stress, or anxiety—and the results revealed complete, partial, and partial mediation in this regard.

Because hearing loss is (1) the only established ontological risk factor for tinnitus symptom onset [15], (2) directly related to age [20], and (3) directly related to tinnitus-related distress [18,41], hearing abilities were of particular interest in this study. Indeed, the results revealed that PTA significantly increased with an increased age and that its effects completely mediated the relationship between age and tinnitus-related distress. Notwithstanding, significant indirect effects of age on tinnitus-related distress via perceived stress and anxiety further highlighted the important role of psychological factors in contributing to or constituting tinnitus-related distress. The psychological dimensions of hearing loss [4,5,6,7,8,9], psychological distress on developing hearing loss [10,11], as well as the role of psychological factors in accepting hearing amplification devices have been well-established [62,63,64]. Psychological factors thus form key targets to prevent or ameliorate both hearing loss, tinnitus-symptom onset, and the likelihood of tinnitus-related distress with increasing age.

Last, we examined how interactions of the examined mediators influenced the effect of age on tinnitus-related distress. PTA was not associated with perceived stress and anxiety. This finding was not in keeping with the previous literature, where hearing loss was associated with stress [10,11] and depression and anxiety [4,5,6,7,8,9,12,13]. However, any such association may have been detected in a clinical sample with higher levels of psychological distress, more severe hearing impairment, or only subjects with hearing loss (PTA > 26 dB [2]).

Perceived stress and anxiety jointly mediated the effect of age on tinnitus-related distress. Construct overlaps between stress and anxiety might explain changes in the direct effect between age and anxiety, compared with the regression analysis and mediation with only one mediator.

The mediation model revealed negative indirect effects when perceived stress was included. In accordance with Zhao et al. [65], this could be interpreted as competitive partial mediation. We therefore assumed that age-related changes in perceived stress might have a buffering effect on the relationship between age and tinnitus-related distress. This effect could be classified in the aforementioned research on resilience in old age [60,61].

Due to the different signs of the direct and indirect effects, according to Zhao, Lynch and Chen [65], it is also likely that the relationship between age and tinnitus-related distress is mediated by another variable that is not included in the model. This in turn supports the finding that an age-related increase in tinnitus-related distress is more likely mediated by hearing abilities, as discussed before.

Overall, the results of the present study suggested that an increased age heightens the risk of hearing loss and associated tinnitus symptom onset. Whilst hearing abilities explained the effect of age on tinnitus-related distress, psychological factors may constitute or protect against tinnitus-related distress in the context of age-related symptom occurrence.

### Limitations

It should be taken into account that the results were based on correlations and that no conclusions on causality can be drawn due to the cross-sectional design. Moreover, the relations could be bidirectional. Other variables that were not included in the model might have influenced the observed effects. With regard to the sample, it should be taken into account that there were only a few participants over the age of 70 years old (*N* = 33), so the results in this cohort were of limited significance and need further investigation. Last, the psychological impact of hearing abilities was not measured directly, and future studies should aim to disentangle associated interactions.

## 6. Conclusions and Clinical Implications

Hearing loss, tinnitus symptom onset, and tinnitus-related distress appeared to increase across the age span. The influence of aging on tinnitus-related distress was fully mediated by hearing ability. Psychological distress appeared to improve with age, thus acting as an ameliorating “buffer” in the relationship between age and tinnitus-related distress. Our results confirmed the complexity and multimodality of chronic tinnitus. Changes in tinnitus-related distress associated with aging were not primarily due to age, but to the effects of auditory perception and psychological factors. This implied that with an increased age, manifestations of hearing loss and its psychological impact should be taken into account when aiming to prevent or treat tinnitus symptom onset or tinnitus-related distress. This is especially important keeping in mind that a decrease in psychotherapy utilization is observed after age 55 [34]. Our findings could help design prevention programs tailored to the specific needs of aging people or facilitate access to psychotherapy through a heightened awareness of these factors. Examining and understanding underlying processes that explain the observed reductions in stress and anxiety with aging could also help identify protective factors in future. Future studies should include stress resilience in older age and its interactions with anxiety and depression.

## Figures and Tables

**Table 1:** Demographic variables.

		Age Group			
Variable					
	Total	Adolescence (<20)	Young Adulthood (20–39)	Middle Adulthood (40–64)	Late Adulthood (>65)
	*N* (%)	*N* (%)	*N* (%)	*N* (%)	*N* (%)
**Total**	678 (100)	1 (0.15)	121 (17.85)	471 (69.47)	85 (12.54)
**Gender**					
Female	347 (51.2)	-	59 (48.8)	244 (51.8)	44 (51.8)
Male	331 (48.8)	1 (100)	62 (51.2)	227 (48.2)	41 (48.2)
**Nationality**					
German	622 (91.7)	1 (100)	98 (81)	440 (93.4)	83 (97.6)
Other	56 (8.3)	-	23 (19)	31 (6.6)	2 (2.4)
**Marital Status**					
Single	159 (23.5)	1 (100)	64 (52.9)	82 (17.4)	12 (14.1)
Partnership	83 (12.2)	-	31 (25.6)	46 (9.8)	6 (7.1)
Married	333 (49.1)	-	24 (19.8)	257 (54.6)	52 (61.2)
Divorced	86 (12.7)	-	2 (1.7)	76 (16.1)	8 (9.4)
Widowed	17 (2.5)	-	-	10 (2.1)	7 (8.2)
**Educational Degree**					
None	6 (0.9)	-	1 (0.8)	4 (0.8)	1 (1.2)
School	225 (33.2)	1 (100)	24 (19.8)	171 (36.3)	29 (34.1)
Apprenticeship	144 (21.2)	-	14 (11.6)	118 (25.1)	12 (14.1)
Polytechnic degree	76 (11.2)	-	8 (6.6)	55 (11.7)	13 (15.3)
University degree	227 (33.5)	-	74 (61.2)	123 (26.1)	30 (35.3)
**Work status**					
Employed	478 (70.5)	-	90 (74.4)	383 (81.3)	5 (5.9)
Unemployed	200 (29.5)	1 (100)	31 (25.6)	91 (18.7)	80 (94.1)

**Table 2:** Means, standard deviation, and group comparisons for the examined variables across the age span. PSQ = Perceived Stress Questionnaire; HADS-A = Hospital Anxiety and Depression Scale—anxiety subscale; HADS-D = Hospital Anxiety and Depression Scale—depression subscale; TQ = Tinnitus Questionnaire; PTA = pure-tone average.

		PSQ	HADS-A	HADS-D	TQ	PTA
	*n*	*M (SD)*	*M (SD)*	*M (SD)*	*M (SD)*	*M (SD)*
Total	678	50.92 (20.2)	8.51 (4.14)	7.07 (4.7)	39.98 (16.32)	19.23 (11.96)
Age group						
<20	1	25 (-)	7 (-)	0 (-)	39 (-)	5 (-)
20–39	121	55.18 (16.97)	8.88 (3.39)	6.1 (3.64)	35.12 (13.87)	9.76 (6.97)
40–64	471	52.68 (20.02)	8.67 (4.23)	7.62 (4.88)	41.75 (16.98)	20.18 (11.44)
>65	85	35.41 (18.45)	7.12 (4.38)	5.54 (4.37)	36.31 (13.91)	27.64 (11.96)
		Welch’s *F*(2, 188.45) = 35.53, *p* < 0.001	Welch’s *F*(2, 185.18) = 5.3, *p* < 0.001	Welch’s *F*(2, 194.9) = 12.46, *p* < 0.001	Welch’s *F*(2, 196.31) = 12.3, *p* < 0.001	Welch’s *F*(2, 195.88) = 116.74, *p* < 0.001

## Data Availability

As per Charité Universitaetsmedizin Berlin’s ethics committee, unfortunately we cannot make the data public without restrictions because we did not obtain patients’ consent to do so at the time. Nevertheless, interested researchers can contact the directorate of the Tinnitus Center Charité Universitaetsmedizin Berlin with data access requests (birgit.mazurek@charite.de). Alternatively, interested researchers may also contact Charité’s Open Data and Research Data Management Officer Dr. Evgeny Bobrov (evgeny.bobrov@charite.de).
